# Evaluation of a consulting training course for international development assistance for health

**DOI:** 10.1186/s12909-018-1339-3

**Published:** 2018-10-11

**Authors:** Pan Gao, Hao Xiang, Suyang Liu, Yisi Liu, Shengjie Dong, Feifei Liu, Wenyuan Yu, Xiangyu Li, Li Guan, Yuanyuan Chu, Zongfu Mao, Shu Chen, Shenglan Tang

**Affiliations:** 1 Department of Global Health, School of Health Sciences, Wuhan University, 115# Donghu Road, Wuhan, 430071 China; 2 Global Health Institute, Wuhan University, 115# Donghu Road, Wuhan, 430071 China; 3 Global Health Research Center, Duke Kunshan University, 8# Duke Avenue, Kunshan, 215316 China; 4 Duke Global Health Institute, Duke University, Trent Hall, 310 Trent Drive, Durham, North Carolina 27710 USA

**Keywords:** Development assistance for health, International consultation, Kirkpatrick’s model, Program evaluation

## Abstract

**Background:**

Development assistance for health (DAH) is an important component of foreign assistance. International health consultants usually play a key role in the international DAH field. However, there is still a shortage of consulting training in China. To address this issue and develop new backup force of DAH for China, the Global Health Institute of Wuhan University (GHIWHU) launched a training program called the “Consulting Training Course for International Development Assistance for Health”. The purpose of this article is to evaluate the impact of the training on participants.

**Methods:**

We conducted the analysis using Kirkpatrick’s model. An evaluation survey examining participants’ reaction (level 1) and learning (level 2) was carried out among trainees following the training, and a follow-up telephone interview of application (level 3) was made in three months after the training.

**Results:**

A total of 25 participants from Chinese Consortium of Universities for Global Health (CCUGH) attended the training program. Results of satisfaction evaluation indicated that the training program was well received, with more than 85% of participants felt satisfied or relatively satisfied with the training. Trainees’ self-ratings of the consulting knowledge and skills showed a significant increase (*p* < 0.001) from pre- to post-training. The follow-up interview revealed that the majority of participants applied the acquired knowledge and skills under various circumstances such as consulting program, teaching processes, writing reports, and et al. Meanwhile, participants considered that the lack of opportunities was one of the major application barriers. In addition, they expressed the willingness to participate in more relevant training and the need for more practice opportunities.

**Conclusions:**

This is the first study evaluating a consulting training program in China. The results show that the training course has been successfully implemented and participants have been given consulting knowledge and skills. Future research should use better-designed training methods based on demand surveys and consider providing participants with practice or practicum opportunities. Also, it is necessary to conduct both primary and advanced training courses and evaluate participants’ long-term behavior changes resulting from the training.

## Background

The 2030 Agenda for Sustainable Development increases focus on global health [1]. Nowadays, China is becoming increasingly influential in the field of global health and has been under-recognized by the international health community [2]. Emerging powers, including China, have gradually become donors of international health assistance [3–5]. According to the reported total amount of foreign aid, the actual amount invested by the Chinese government on Development assistance for health (DAH) was the highest among the BRICS countries during 2005–2010, reaching a peak of about $550 million in 2010 [6]. Additionally in December 2015, the Chinese government agreed to spend $60 billion in three consecutive years to boost its cooperation with Africa that would benefit 10 major projects, including China-Africa public health cooperation program [7]. These commitments and initiatives reflected the willingness of China to provide more international assistance and make greater contributions to the international community. Meanwhile, the developed countries in Europe are also concerned about the construction of global health value system [8]. To handle the global health challenges, more actions are needed for workforce planning and capacity building of health professionals [9]. As one of the emerging powers, Brazil is also actively involved with global health issues to enhance its international influence [10, 11]. However, China’s efforts in global health have not kept up with the pace of other countries, especially for the training of global health consultants.

Having qualified global health professionals is a prerequisite for any country’s involvement in global health. Faced with many challenges in public health, complex methods and strategies need to be designed by qualified public health workers to make effective responses [12]. With the strengthening of international responsibilities, China has established solid relations with the African continent over the past three decades by providing all kinds of assistance to Africa, including those in the field of health [13]. Ministerial Forum on China-Africa Health Development, one of various China-Africa health-related cooperation programs, has been established to strengthen health cooperation between China and Africa [14]. In addition, the Africa Centres for Disease Control and Prevention (Africa CDC), supported by the Chinese Centers for Disease Control and Prevention (CCDC) and the United States Centers for Disease Control and Prevention (US CDC), has been launched to address Africa’s health problems [15]. However, there is a huge gap between the capabilities of Chinese government officials and experts in international health programs and the needs of global health projects. Compared with the developed countries, China’s professionals engaging in global health mainly come from government, universities, and relevant research institutes. This makes it difficult to apply Chinese experiences to the African context [16]. Chinese personnel are unable to provide high quality health advisory services and most of them lack practical experience. Few people have the skills to provide consultation. To a certain extent, this has limited China’s further participation in the field of global health.

To address this challenge, a training program, “Consulting Training Course for International Development Assistance for Health”, has been implemented by the Global Health Institute of Wuhan University (GHIWHU) under the support of Chinese Consortium of Universities for Global Health (CCUGH). The theme of this training is “how to provide technical advisory services to the international health development assistance program”. The training objects are experts, scholars, government personnel, and a few college students from the CCUGH member units. This training program is designed to: (1) enhance trainees’ understanding of global health, international DAH and the role of consultants; (2) help them recognize the whole process of technical consultation; (3) help participants acquire fundamental consulting knowledge and skills. At present, the training program of DAH for consultants has just emerged in China. Additionally, evaluation is an essential step in the building of a curriculum or program [17–20]. As one of the earliest training courses in China, it is necessary to conduct an evaluation of the training program [21]. This paper describes the process and evaluation of the training, including satisfaction assessment, self-ratings of knowledge and skills, and a follow-up interview of application after 3 months of the course. To our knowledge, this is the first study to present the impact of a consulting training program on trainees in China.

## Methods

### Study design

The research was conducted by a mixed-methods approach based on Kirkpatrick’s model, using both quantitative and qualitative data. Participants received an evaluation questionnaire regarding their satisfaction of the training program and retrospective self-assessments on knowledge and skills by email after the training. A semi-structured telephone interview was adopted in the follow-up study to investigate trainees’ application on the acquired knowledge and skills after three months of the training.

### Participants

We released the information regarding this training on CCUGH website on August 8, 2016. Participants registered the training program through the Mymova network system. As a result, a total of 25 applicants from universities, national health development research departments, and CCDC were recruited into the program. The study was approved by the Ethics Committee of Wuhan University School of Medicine. We obtained trainees’ oral informed consent before implementing both the training evaluation questionnaire and follow-up interview. In addition, the written informed consent was included in the cover letter of the questionnaire and sent to each participant.

### The training course

Training content and teaching methods were designed carefully based on necessary competences in the international DAH consultation program. Through several rounds of discussion and debates, a consensus on the final arrangements and detailed contents of the training course was reached. The two and a half-day training program was implemented at School of Health Sciences, Wuhan University, between October 9 and October 11, 2016.

The training courses comprised 11 sessions, divided into 3 modules including theory, methods, and practice. It is noteworthy that the distance learning was used during the 6th session and the rest were all on-site training courses (see Table 1). Methods of interactive learning between trainers and participants were applied throughout the training. Combined with traditional didactic approach, various training methods were used including group discussion, case analysis, simulation training method, and role-play. The duration for each session was one and a half hours. Under normal circumstances, half an hour would be set aside in each session for trainees to express their thoughts and ask questions. Trainers would then respond to these questions and make appropriate adjustments to the course schedule.

**Table 1:** Course guide of the consulting training program

Modules	Sessions	Teaching Approach
Theory	1. International DAH	On-site training
	2. Global health	On-site training
	3. Consulting preparation and grouping	On-site training
	4. ToR&CVs	On-site training
Methods	5. analysis method of health system	On-site training
	6. role of consultants	Distance learning
	7. methods of collecting and analyzing data	On-site training
	8. Consultation plan	On-site training
Practice	9. Implementation of consulting activities	On-site training
	10. Writing consulting report	On-site training
	11. Simulation training and group reporting	On-site training

### Evaluation model

We applied Kirkpatrick’s model to evaluate the outcomes of the training program [22]. As a standard assessment tool, this model was widely used for training program evaluation [17, 23, 24]. Additionally, it can provide a systematic evaluation framework as both quantitative and qualitative data were collected during the assessment [25]. The model focused on four levels of outcomes: (1) reaction of participants (the satisfaction of participants during the training); (2) participants’ learning (attitude changes of participants, their knowledge and skills obtained from the training); (3) behaviors of participants (applying knowledge and skills during daily practice); (4) the overall results (the impact of the training on the organization or institution). As the four levels are not ordered hierarchically, this model provided a flexible structure that can be tailored according to the needs of a specific evaluation [26]. The aim of this study was to evaluate the effect of a training program on participants’ individual development, and we mainly focused on the first three levels.

### Assessment tools and measures

After the training, participants were asked to complete a two-section training assessment questionnaire. The first section was a satisfaction evaluation test and was used to assess participants’ reaction on the training. Evaluation indicators included the satisfaction of course content, teaching methods, trainers, and program organization. Participants were asked to rate these indicators on a 5-point Likert scale, ranging from “unsatisfied” (1) to “satisfied” (5). The usefulness of the training and their willingness to participate in consulting services were also surveyed. Additionally, participants could write suggestions on the training in a free-text comment box. The second section was a retrospective knowledge and skills test, which was comprised of 25 items in eight modules (see Table 3). We asked participants to assess their learning of the training using a self-efficacy survey which was based on a 5-point Likert scale ranging from “know nothing about it” (1) to “can apply knowledge and skills effectively” (5). Combined with the advice from training experts, the evaluation model and the course content, the questionnaire was designed by GHIWHU researchers. Prior to the actual evaluation, the questionnaire was distributed to the volunteers from the CCUGH as a pilot study. The final version of the questionnaire was determined after several rounds of pilot investigations.

After searching literature and adopting ideas from instructors, we determined the interview guide before the follow-up interview. To evaluate the application of knowledge and skills, we interviewed participants who consented to participate in the follow-up assessment approximately three months after the training. The telephone interview consisted of five open-ended questions in three parts, including the daily application of the learning, application barriers and further learning. For instance, we asked participants to describe their daily practice (e.g., Have you applied the acquired knowledge in your daily work and study? If so, please give examples). In addition, we encouraged participants to report barriers in the application of the acquired knowledge and skills. The interview also explored participants’ willingness to attend more training and their expectations on further training. In order to assess the interview guideline and help interviewers master the interview skills, face to face interviews based on the impact of the training were conducted within the Global Health Institute.

### Data collection and analysis

We extracted the baseline data (gender, age, title, position, and e-mail address) from the registration system and managed them in Microsoft Excel 2010. We also downloaded resumes of participants. Within one week after the training, all participants were surveyed anonymously. Finally, we collected 23 questionnaires, with 21 of them being valid and the other 2 uncompleted. Meanwhile, each participant was informed that a telephone interview would be conducted to access the long-term effect of the training. We consented that the information collected from interviews would be kept confidential. As a result, 22 trainees completed their interviews. All interviews were audiotaped, and were transcribed verbatim into the Microsoft word 2010 text format.

EpiData 3.0 was used to establish databases, and SPSS 19.0 was used for statistical analysis. Quantitative data (baseline data and training satisfaction ratings) were descriptively analyzed. Because of the relatively small sample, the results of self-assessments in pre- and post-training were not normally distributed. Therefore, median, upper quartile, lower quartile, and non­parametric statistics were used in the analysis. The Wilcoxon signed ranks test was used to compare the self-assessments of knowledge and skills before and after the training. A *p* value less than 0.05 was considered statistically significant. Missing data (uncompleted questionnaires) were excluded from the analysis.

Qualitative data (text comments and telephone interview contents) were analyzed using content analysis [27]. The first author initially developed a code list according to the interview guide. Then, two authors read each transcript carefully and categorized the text data individually. The first author subsequently compared the themes by checking differences and similarities of the results. During this process, codes were combined or split in order to ensure a more accurate categorization. When a disagreement on the code emerged, the two authors would discuss the difference to reach a consensus. All responses would be reviewed by both authors in the end.

## Results

### Respondent demographics

Twenty-five participants took part in the training course. These responders have different occupations: academics (9), civil servants (3), researchers (4) coming from health departments of government, and communication and advocacy officer (1) coming from the Joint United Nations Programme on HIV/AIDS (UNAIDS) China focal point. The rest of participants (*n* = 8) were medical students. The majority of participants were females (71.4%), with an average age of 30 years. The participants have good education backgrounds. More than half of them (*n* = 15, 60%) earned a master’s or doctoral degree. In addition, the participants expressed strong interests in international DAH. A total of 21 participants completed the training evaluation survey, and 22 completed the follow-up interview. The response rates were 84% and 88%, respectively.

### Satisfaction evaluations

The results of satisfaction evaluations showed the training was well received. More than 95% of participants responded that they were relatively satisfied or satisfied with the training scheme, and the satisfaction rate was 100% on training environment. More than 85% of participants were satisfied with course content, teaching methods, and trainers (see Table 2). In addition, 8 participants (38.1%) responded that the acquired knowledge of the training would be probably useful in their future work, while 12 (57.1%) thought it would be certainly helpful to their work. Moreover, when trainees were asked “If there is an opportunity, would you like to take part in the international consulting work”, 14 (66.7%) responded that they would certainly engage in the work. Content analysis of the free-text comment responses revealed further information regarding training assessment. Participants indicated that the group homework helped apply the acquired knowledge and skills in practice effectively, as “*enthusiasm for learning will be enhanced under stresses between groups*”. Some participants advised that enrollment publicity of the training should be strengthened, because “*I hope more young scholars would have the opportunity to participate in such good training*”. Meanwhile, more than one participant expressed that the effect of distance learning was not good. Also, participants expected to add interactive activities in small groups to help with the mutual acquaintance and communication in the future.

**Table 2:** Participants’ satisfaction evaluation of the training courses

Courses modules	The number of participants scored 4 and 5^a^ (%)		
	Course content	Teaching methods	trainers
International DAH	19(90.5)	19(90.5)	19(90.5)
Global health	18(85.7)	18(85.7)	18(85.7)
ToR & CV	17(94.4)*	17(94.4)*	17(94.4)*
Analysis method of health system	19(90.5)	20(95.2)	21(100.0)
Role of consultants	19(90.5)	18(85.7)	20(95.2)
Methods of collecting and analyzing data	18(85.7)	19(90.5)	19(90.5)
Organization and implementation of consulting activities	21(100.0)	21(100.0)	21(100.0)
Writing consulting report	19(90.5)	19(90.5)	19(90.5)

^a^Rating scale: 1 = unsatisfied, 2 = relatively unsatisfied, 3 = modest, 4 = relatively satisfied, 5 = satisfied

*There were three participants who didn’t attend this course

### Knowledge and skills assessment

Table 3 shows results of pre- and post-training self-assessment of trainees on knowledge and skills. The Wilcoxon’s signed ranks test indicated a significant improvement in the post-training assessment for all 25 items in eight modules, as compared with the pre-training evaluation (*p* < 0.001). Prior to the training, the self-rated knowledge and skills scores regarding the eight modules were relatively low, ranging from 1 to 4. After the training, the improvement was observed that all items’ scores reached 3 and higher. More than 75% of participants obtained some knowledge and over half of them grasped relevant knowledge and skills on the consulting service after the training. A higher gain in self-assessed knowledge and skills could be found in the modules: Terms of Reference (ToR) & Curriculum Vitae (CV); organization and implementation of consulting activities; and writing consulting report. The items for the concept of global health, Global health governance, six elements of the health system, epidemiological analysis methods, and quantitative research methods have been marginally increased. It is noteworthy that in the 25 self-ratings, 7 items’ scores reached 4.5 or above, including definition of international DAH, quality and ability of experts, project management, six elements of the health system, epidemiological analysis methods, China’s contribution to global health, and quantitative research methods.

**Table 3:** Self-assessment on knowledge and skills for pre-and post-training course

Course modules	Consulting knowledge/skills	Pre-	Post-	Z-value
		M(*P*_25_~*P*_75_)^a^	M(*P*_25_~*P*_75_)^a^	
(1) International DAH	Definition	3(2~ 3)	4(4~ 4.5)	− 3.684
	Aid agencies and organizations	2(2~ 3)	4(3.5~ 4)	− 3.923
	Achievements and challenges	3(2~ 3)	4(3.5~ 4)	−3.825
(2) Global health	Concept	3(3~ 4)	4(4~ 4)	−3.491
	Global health governance	3(2~ 4)	4(3.5~ 4)	−3.520
	Health diplomacy in China	3(2~ 3.5)	4(3~ 4)	−3.640
	Quality and ability of experts	2(1~ 3)	4(3~ 4.5)	−3.926
	Project management	3(1.5~ 3)	4(3~ 4.5)	−3.852
(3) ToR & CV	Components of ToR	2(1~ 2)	4(3.5~ 4)	−3.785
	Writing and modifying CV	2(1.5~ 3)	4(3.5~ 4)	−3.682
(4) Analysis method of health system	Six elements of the health system	3(2~ 4)	4(4~ 4.5)	−3.660
	Epidemiological analysis methods	4(3~ 4)	4(4~ 4.5)	−3.000
(5) Role of consultants	Global health challenges	3(3~ 3)	4(4~ 4)	−3.640
	China’s contribution to global health	3(2~ 3)	4(3~ 4.5)	−3.739
	China participates in global health consultation	2(1.5~ 3)	4(3~ 4)	−3.874
(6) Methods of collecting and analyzing data	Design discipline	3(2.5~ 3)	4(3.5~ 4)	−3.945
	Scientificalness and feasibility analysis	3(2.5~ 3)	4(3.5~ 4)	− 4.146
	Quantitative research methods	3(3~ 4)	4(3~ 4.5)	−3.771
	Qualitative research methods	3(2~ 3.5)	4(3~ 4)	−3.704
	Similarities and differences between consultation and research	2(1~ 3)	4(3.5~ 4)	−3.867
(7) Organizing and implementing the consulting activities	Consulting preparation	2(1~ 3)	4(3~ 4)	−4.072
	Implementing consultation	2(1~ 3)	4(3~ 4)	−4.075
	Completing of consultation	2(1~ 3)	4(3~ 4)	−4.086
(8) Writing report	Structure and content	2(1~ 3)	4(3~ 4)	−4.064
	Writing cautions	2(1~ 2.5)	4(3~ 4)	−4.083

^a^Rating scale: 1 = know nothing about it, 2 = have heard something about it, 3 = have some knowledge but no skills, 4 = have the relevant knowledge and skills, 5 = can apply knowledge and skills effectively; “M” = median, “*P*_25_” = upper quartile, “*P*_75_” = lower quartile; all of the *p* values < 0.001

### Follow-up interview

A total of 22 participants (88%) received the telephone interview. Table 4 shows the results on applying the acquired knowledge and skills in the follow-up interviews. 19 participants (86.4%) had applied the acquired knowledge and skills during their daily work or study (including teaching processes, writing reports, collecting and analyzing data, and updating CV), while only one participant applied them in the consulting program. Besides, two participants have not applied the acquired knowledge yet. Some of the participants indicated that:

**Table 4:** Results of applying the acquired knowledge and skills in the follow-up interview

major topics	*N/*22
Application of the acquired knowledge and skills	
Applied to daily work or study	19
Applied to the consulting program	1
Haven’t applied	2
The most influential training knowledge and skills on daily practice	
All of it was useful	12
Theoretical knowledge of international Development Assistance for Health consultant	12
Methods of collecting and analyzing data	10
Writing ToR & CV	9
Share the knowledge of training	
Shared with colleagues or classmates	18
Haven’t shared	3
Application barriers of Training knowledge and skills	
No opportunity	20
No appropriate consulting program	7
Need self-improvement	6
Further training and learning	
If there are opportunities, I’m glad to attending more training	21
Hope to add more consulting cases	16
Hope to participate in consulting practice	7
Won’t attend similar training	1

*“I used it in my work. In the process of course teaching, I introduced the theory about global health to my students.”*

*“The theoretical part was useful when I wrote research reports, including the role of international development assistance for health, problems China is facing, and the function of World Health Organization (WHO).”*

When participants were asked about the impact of training on their day-to-day practices, they responded that it was difficult to articulate the concrete aspects of the most and least influential parts in the training and more than half of them considered all the acquired knowledge to be useful. Specifically, trainees applied knowledge and skills, namely, theoretical knowledge of international DAH consultant, methods of collecting and analyzing data, and writing ToR & CV most frequently. Except for 3 participants, all of the trainees have shared the acquired knowledge and experience with their colleagues or classmates. The following are some responses from the participants:

*“In the global health center of Zhejiang University, I shared the training information with teachers, doctors, and post-graduates at the regular meeting.”*

*“That evening, I came back from training, and I immediately talked to students about what I have learned from the training.”*

In addition, participants also encountered obstacles when applying these knowledge and skills. Most of them said they did not have the opportunity to participate in the international consultation program. Some said that there is no suitable consulting program for them before they could get improved. In terms of further training, 95.5% of the participants (*n* = 21) expressed that they were glad to attend more training. Only one trainee responded: “*I wouldn’t attend such training in the near future. I attended another training in Beijing after this training. Now I am waiting for the practice opportunities to use knowledge I learned from these two training courses.”* About suggestions, more consulting cases discussions and consulting practices were expected. Some of the trainees indicated:

*“More cases that the trainer has completed should be added. I want to learn every step of the case and more detailed operations”.*

*“Different forms of training or activities tailored to different needs should be designed. If our practicum regarding consulting activities cannot be conducted outside the country, it may be more effective to have an interview or a seminar in a practicum such as half-day or two hours in the domestic sector. I hope to practice.”*

## Discussion

In this research, we conducted an evaluation of an international DAH consulting training that aimed to enhance participants’ technical consulting competence. The evaluation results revealed that participants were satisfied with the training program and their self-assessment showed a significant improvement of their consulting knowledge and skills upon completion of the training. According to the follow-up interview, participants have applied the knowledge and skills they learned in their daily work and study. To our knowledge, this is the first study to evaluate an international DAH consulting training in China.

Previous studies have demonstrated that characteristics of participants may affect the training outcomes. Frantz et al. found [28] that the diversity of experiences, nationalities, culture and professions, and present in participants’ recruitments strongly influenced the personal development of trainees and group identity in training sessions. Harris et al. found the training program could be strengthened through interprofessional dialogue between multiple professionals during the training [29]. In this study, although the participants came from different backgrounds, the participants’ engaging fields were relatively similar. As international DAH consulting work are multidisciplinary, cross-cultural and cross-regional, WHO attaches great importance to cross-professional education and cooperative practices around the world [30]. Therefore, it is essential to train substantial numbers of experts with different professions, diverse educational backgrounds, and rich experience to bolster the global health workforce.

In the satisfaction assessment, participants gave a high rating on all aspects of the training program. Almost all the participants were satisfied, which could be interpreted as trainers with extensive consulting experience in the international DAH field and trainees were interested in the training content. Furthermore, a previous study showed that a high level of participants’ satisfaction was important for projects that helped to prevent a decline in participation [31]. In addition, participants thought the training was useful and some of them were willing to attend DAH consulting program in future, which showed the training was successful and meaningful. Prior studies also found that changes of participants’ attitudes and behavioral intentions could influence their knowledge and performance during and after the training [32, 33].

The issue of distance learning is worth noting. With the progress of computer technology, a large number of training courses adopted the way of network learning and achieved good results [34, 35]. In this research, results of qualitative responses showed that distance learning was less useful than other methods in imparting consulting knowledge. Several factors may explain this phenomenon. First, the course taught by distance learning was in English and the trainer spoke at a relatively fast speed. For non-native English speakers, language is one of the major barriers for learning [36]. To address this problem, Brownson et al. suggested [37] that it was essential to have clear slides, to keep presentations at a reasonably slow pace, and to allow plenty of time for questions in the course. Second, Internet connection and modern information technology had an impact on the quality of distance learning [35, 38]. Third, the lack of interaction between trainers and distance learners may be another reason for this phenomenon. Existing research shows that trainers and trainees prefer interactive teaching, which could usually receive good results during the training and is useful for participants to obtain knowledge and skills [17, 39–41].

According to the consulting knowledge and skills assessment results, there was a significant increase in participants’ self-ratings. After the training, the scores for all items were increased to greater than 3. This may be because that trainees are highly educated (more than half of them hold masters’ or doctoral degrees) and possess a background in public health. Similarly, Maxwell et al. found [42] that trainees with high levels of education tend to enhance self-efficacy following the training. A previous study also pointed out that personal efficacy is influenced by the experiential sources and the self-efficacy can be enhanced by the mastery experiences of the related and non-related areas [43]. Furthermore, most of participants are young scholars that are open to new knowledge and re-education. In addition, few participants’ self-scores reached close to 5 (can apply knowledge and skills effectively) in a few sessions of the training. This may be explained by the fact that the investigation was conducted within a week after the training and it was difficult for participants to immediately apply the acquired knowledge and skills to their work. This is similar to results found by Silva et al. [44]. Besides, most of participants were new to the field of DAH making them difficult to apply knowledge and skills proficiently.

The study found that most trainees applied the acquired knowledge and skills in various aspects, while only one applied them in the international consulting program. Lack of opportunity for consulting practice was the biggest barrier leading to this outcome. Several key factors may account for this situation. First, DAH consulting training is not yet mature in China and lacks the platform for clients and consultants to share information between each other. Second, some experienced trainers indicated that clients, such as WHO project officers, have their own think tanks. Third, consistent with findings of Ridde et al. [17], it is relatively easier to acquire knowledge than to apply them.

The participants expressed their training needs for further learning. Almost all of them responded that they were glad to attend more training courses. They also hope that future training can add more consulting cases, and training courses can be divided into ones with introductory- and advanced-levels. Regarding the form of training, some participants suggested that the training sponsors could organize practicum in the domestic sector. It is also worth noting that the participants considered that the training program successfully introduced the panoramic view of international DAH consulting knowledge and broadened their horizons.

### Limitation

Several limitations exist in the research. First, this training recruited members restricted to CCUGH leading to a small sample size and the reduced statistical power. Future training should recruit more trainees who are interested in engaging in international DAH programs. Second, we did not conduct the demand survey for training content and methods. Even though, the satisfaction evaluation results indicated that participants gave a high rating on the training. We will make improvements regarding this issue and conduct a needs assessment in our next training course. Third, Due to the lack of appropriate testing tools, we had to use self-assessment questionnaires rather than objective assessments to evaluate the results. Finally, we did not complete the fourth level of the Kirkpatrick model because it is very difficult to assess the long-term impact of the training. And various factors can influence the training effectiveness, such as organizational environment [38, 45].

## Conclusion

The consulting training of international DAH in China is still in its infancy. This is the first study to present an evaluation of a consulting training program carried out in China. The results of this study show that the training has been well received and we have successfully introduced the participants to the initial procedures for the consulting activities. Also, the research demonstrated that a short-period training of international DAH could be used as a tool to improve the consulting knowledge and skills of health professionals. Compared to distance learning, some trainees prefer on-site training that is more effective. Furthermore, most participants commented that they need similar training with more consulting cases, and the lack of access to consulting practice was the biggest barrier. This study provides a useful consulting training model for international DAH in China. Additionally, it is necessary to provide more primary and advanced training courses to evaluate participants’ long-term behavior changes resulting from the training.

## Abbreviations

Africa CDC: Africa Centres for Disease Control and Prevention
BRICS: Brazil, Russia, India, China, and South Africa
CCDC: the Chinese Centers for Disease Control and Prevention
CCUGH: Chinese Consortium of Universities for Global Health
CV: Curriculum Vitae
DAH: Development assistance for health
GHIWHU: Global Health Institute of Wuhan University
ToR: Terms of Reference
UNAIDS: the Joint United Nations Programme on HIV/AIDS
US CDC: the United States Centers for Disease Control and Prevention
WHO: World Health Organization
